# Impact of Sex-Related Differences in Infrarenal Aortic Neck Morphology on Outcomes of Endovascular Aneurysm Repair for Similar-Sized Aortic Aneurysm

**DOI:** 10.3390/diagnostics15020157

**Published:** 2025-01-12

**Authors:** Ombretta Martinelli, Antonio Marzano, Maria Irene Bellini, Roberto Gattuso, Luca Di Marzo, Valeria Gonta, Jihad Jabbour, Wassim Mansour, Simone Cuozzo

**Affiliations:** 1 Vascular Surgery Division, Department of Surgery “Paride Stefanini”, Policlinico Umberto I—“La Sapienza” University of Rome, Viale del Policlinico, 00161 Rome, Italy; ombretta.martinelli@uniroma1.it (O.M.); roberto.gattuso@uniroma1.it (R.G.); luca.dimarzo@uniroma1.it (L.D.M.); valeriagalasan@yahoo.com (V.G.); drjihadjabbour@gmail.com (J.J.); wassim.mansour@uniroma1.it (W.M.); simone.cuozzo89@gmail.com (S.C.); 2 Department of Surgery, Policlinico Umberto I—“La Sapienza” University of Rome, Viale del Policlinico, 00161 Rome, Italy

**Keywords:** abdominal aortic aneurysm, endovascular repair, gender-related aortic neck anatomy, neck-related outcomes, off-label EVAR

## Abstract

**Objectives**: This study aimed to evaluate whether gender-related anatomical differences in proximal aortic neck morphology affect the feasibility and outcomes of endovascular aortic aneurysm repair (EVAR) in women with abdominal aortic aneurysms (AAA). **Methods**: This study performed a retrospective analysis of patients electively treated by EVAR for infrarenal AAA between January 2019 and December 2023. Demographics, anatomical characteristics, and stent graft details were analyzed. The primary endpoint was technical success. Secondary endpoints included freedom from aortic and neck-related reinterventions, endoleak rate, and freedom from aneurysm-related mortality during follow-up. Technical aspects, including adherence to the instructions for use (IFUs), were retrospectively analyzed. **Results**: One-hundred-seventeen patients (fifty-six females; mean age 76.2 ± 5.3 years) underwent elective EVAR for AAA. Demographics and comorbidities were homogeneous across genders. Female patients (Group A) demonstrated a higher prevalence of hostile proximal aortic neck features, including neck length < 10 mm and angulation > 60° (*p* = 0.009, *p* = 0.029, respectively) and a higher frequency of off-label EVAR procedures (28.6% vs. 11.5%; *p* = 0.034). The overall technical success rate was 98.3%, with no significant differences between genders in terms of stent graft selection, use of suprarenal fixation, or incidence of type 1–3 endoleaks. The median follow-up period was 35.2 ± 12.7 months, showing comparable rates of neck-related reinterventions, open conversions, and aneurysm-related mortality between genders. Notably, off-label EVAR was identified as an independent risk factor for type 1A endoleaks, reinterventions, and aneurysm-related mortality (*p* < 0.00001, *p* < 0.0001, and *p* = 0.001, respectively). **Conclusions**: Female patients undergoing EVAR often present with hostile proximal aortic neck features and are treated at an older age than males. Despite these differences, technical success rates and mid- to long-term outcomes were comparable between genders, with no variation in stent graft selection or suprarenal fixation use. Effective procedural planning, device selection, and surgical expertise appear to mitigate historical gender-related anatomical challenges. Further large-scale studies are needed to confirm whether anatomical factors alone drive outcomes, irrespective of gender.

## 1. Introduction

Endovascular aneurysm repair (EVAR) has become the standard practice for the treatment of abdominal aortic aneurysm (AAA), especially in high-risk and frail patients with standard anatomy [1]. The wide variety of stent grafts now available on the market allows vascular surgeons to select the most appropriate stent graft tailored to the patient’s anatomy.

However, several studies have demonstrated that females tend to present with more complex AAA geometries compared to males, including smaller aortic and iliac axis diameters, a larger maximum-to-normal diameter ratio, and a wider lumbar vertebral curvature [2]. The complexity of AAA geometries tends to increase with aneurysm size, potentially resulting in hostile proximal aortic neck features and juxta- and suprarenal aortic involvement [3,4]. These gender-related anatomical differences may explain the reduced on-label applicability of stent grafts in female patients [5,6,7].

Indeed, a hostile proximal aortic neck places the EVAR procedure outside the instructions for use (IFUs) of the stent grafts and significantly increases the risk of both early and delayed complications and the need for reinterventions [8,9,10,11].

We aim to assess whether gender-related differences in the proximal aortic neck morphology, with similar AAA sizes, influence the applicability and outcomes of EVAR in women.

## 2. Materials and Methods

Patients undergoing elective standard EVAR for infrarenal AAA between January 2019 and December 2023 were included in this single-center retrospective cohort study.

Clinical, technical, and follow-up data were collected and entered into a specifically maintained database.

The indications for the treatment of AAA were based on the current adopted guidelines [1]. A rapid growth (≥10 mm/year) of the aneurysm was considered a reasonable criterion for intervention in case of maximal transversal diameter < 50 mm.

Patients were divided into two groups based on gender: Group A (female) and Group B (male).

The anatomical characteristics of the proximal aortic neck, rates of suprarenal fixation, use of aortic cuffs, aortic neck-related complications (e.g., type 1A endoleak), aneurysm-related reinterventions, open conversions, and aneurysm-related mortality were recorded and categorized by sex. The rate of off-label EVAR was also assessed.

Cases with missing data for these variables were excluded from the analysis. The terms ‘sex’ and ‘gender’ are used interchangeably to refer to males and females.

The suitability of aortic neck anatomy for EVAR was retrospectively evaluated by reviewing patients’ computed tomography angiography (CTA) images using dedicated software with multiplanar reconstructions (OsiriX MD; Pixmeo SARL, Bernex, Switzerland).

Aortic diameters were measured on axial reconstructions perpendicular to the largest diameter to avoid overestimation caused by vessel tortuosity. The infrarenal aortic neck length was defined as the point where the aortic diameter first showed a 10% increase compared to the diameter at the most caudal renal artery. Aortic neck angulation was measured as the maximum angle observed in all views between the proximal aortic neck and the longitudinal axis of the aneurysm. A hostile proximal aortic neck was defined by the presence of one or more of the following characteristics: neck length < 10 mm, angulation > 60°, diameter at the proximal sealing zone > 28 mm, conical shape, or circumferential calcium or thrombosis exceeding 50% at the proximal sealing zone [12].

For analysis of adherence to the manufacturer’s instructions for use (IFUs) regarding proximal neck anatomy, procedures were classified as off-label if at least one anatomical parameter violated the manufacturer’s criteria for the specific device.

Standard EVAR procedures were performed using the following devices: Endurant II and Endurant IIS (Medtronic, Santa Rosa, CA, USA), Gore Excluder and Gore Conformable (W.L. Gore and Associates, Flagstaff, AZ, USA), Zenith Alpha (Cook, Bloomington, IN, USA), Incraft (Cordis Corp, Bridgewater, NJ, USA), Ovation, Alto and AFX (Endologix, Irvine, CA, USA).

In accordance with reporting standards, technical success was defined as the successful introduction and deployment of the device without the need for additional or secondary surgical or endovascular procedures, death, type 1 or 3 endoleaks, or graft limb occlusion within 24 h post-procedure.

Aortic neck-related mortality was defined as death resulting from AAA rupture after EVAR, complications from secondary procedures, or surgical conversion [13,14]. The follow-up protocol consisted of Duplex ultrasound (DUS) or contrast-enhanced ultrasound (CEUS) examinations at 1, 3, 6, and 12 months post-procedure, followed by annual examinations thereafter. A CTA scan was performed at 1 month and subsequently only if DUS or CEUS detected endoleaks or a significant change in the residual AAA sac size (≥5 mm).

The two groups were compared using the Chi-square or Fisher’s exact tests for unordered categoric factors and Student’s *t*-test for ordinal measures. A univariate analysis using Cox regression modeling was conducted to evaluate whether off-label EVAR procedures were more prone to complications and to identify potential sex-related differences in these complications. A descriptive analysis of all variables was performed. SPSS (v. 27; SPSS Inc., Chicago, IL, USA) and Excel (v.16.89.1 Microsoft Corporation, Redmond, WA, USA) were used for statistical analysis. A *p*-value < 0.05 was considered statistically significant.

The study was conducted according to STROCCS 2021 guidelines [15] and complies with the Declaration of Helsinki [16]. Informed written consent of patients was obtained. Patients gave their consent for publication. Institutional review board approval was requested and obtained.

## 3. Results

One-hundred-seventeen patients (fifty-six females, 47.9%) with a mean age of 76.2 ± 5.3 years underwent elective standard EVAR for infrarenal AAA at our University Hospital Center between January 2019 and December 2023. Patients’ demographics are listed in Table 1. Based on gender, patients were divided into two groups. Fifty-six patients (47.9%), with a mean age of 78.2 ± 2.6 years (range, 73–84), were included in Group A (females), whereas sixty-one patients (52.1%), with a mean age of 74.3 ± 6.4 years (range, 62–87), were included in Group B (males). The demographic characteristics and preoperative major comorbidities were homogeneous between the two groups and not correlated with gender, except for age and the prevalence of peripheral artery disease (*p* = 0.01, 0.012, respectively; Table 1). The median maximal transversal aortic diameter (DT) was 56.7 ± 5.2 mm (range, 50–78). The median AAA diameter was 56.0 ± 5.6 mm in Group A and 57.4 ± 1.9 mm in Group B (*p* = 0.06). The anatomical characteristics of the proximal aortic neck are reported in Table 2. Hostile proximal aortic neck features were observed in 35 patients (25 females vs. 10 males; 44.6% vs. 16.4%, *p* = 0.001). Moreover, a neck length < 10 mm was observed in 30.4% of females and 9.8% of males (*p* = 0.009), while neck angulation > 60° was found in 21.4% of females and 6.6% of males (*p* = 0.029). A neck diameter at the sealing zone > 28 mm was observed in 10.7% of females and 3.3% of males (*p* = 0.15), without reaching statistical significance, as conical shape and calcification/thrombosis > 50% (Table 2). Multivariate analysis examining the association with hostile proximal aortic neck features revealed that females had a greater prevalence of these characteristics compared to men (13/56, 23.2% vs. 5/61, 8.2%, *p* = 0.038).

The type of stent grafts, rate of suprarenal fixation, and off-label use of stent grafts are summarized in Table 3. Twenty-three patients (19.6%) underwent off-label EVAR due to unfavorable proximal aortic neck characteristics; female patients were more prone to off-label EVAR compared to men (28.6% vs. 11.5%; *p* = 0.034).

The overall technical success rate was 98.3%. Two type 1A endoleaks were detected at the completion of angiography. One patient (Group B) underwent an adjunctive procedure with aortic cuff placement to ensure optimal proximal aortic sealing, whereas the other patient (Group A), who was treated with an Ovation stent graft, demonstrated spontaneous sealing of the endoleak on the one-month follow-up CTA scan and CEUS. Percutaneous EVAR was performed in all cases, with no groin complications reported. No statistically significant differences were observed between the groups regarding the choice of stent grafts, the number of proximal aortic cuffs used, and the stent grafts with suprarenal fixation (46.4% vs. 31.1% *p* = 0.12).

The median follow-up time was 35.2 ± 12.7 months (range, 9.8–60.1). Five patients from Group A and six from Group B were lost during the follow-up. Seven patients from Group A (13.7%) required a neck-related reintervention during the follow-up period. Four of them underwent a relining with a proximal aortic cuff, which occurred at 24, 28, 29, and 31 months after the index procedure. Three patients underwent open conversion. The freedom from aneurysm-related mortality in Group A was 96.1%. Four patients from Group B (7.3%) required a neck-related reintervention during the follow-up period. One of them underwent a relining with a proximal aortic cuff, whereas three patients underwent open conversion. The freedom from aneurysm-related mortality in Group A was 96.4%. No significant differences were observed in late type 1A endoleak, neck-related reintervention and open conversions between the two groups (Table 4), despite the greater proportion of off-label EVAR performed in the female cohort (*p* = 0.034).

Statistical analysis confirms that off-label EVAR in an independent risk factor associated with a greater incidence of type 1A endoleaks, neck-related reinterventions, and aneurysm-related mortality in both genders (*p* < 0.00001, <0.0001, and 0.001, respectively).

### Tables

**Table 1:** Demographic characteristics of the patients and distribution according to gender. CAD: coronary artery disease; COPD: chronic obstructive pulmonary disease; PAD: peripheral artery disease; ASA (American Society of Anesthesiologists) score III: patients with significant functional limitations with at least one moderate to severe disease.

Demographics	Overall Population (N. = 117)	Group A (Females) (N. = 56)	Group B (Males) (N. = 61)	*p*-Value
Age (years ± SD)	76.2 ± 5.3	78.2 ± 2.6	74.3 ± 6.4	0.01
Hypertension (*n*, %)	83 (70.9)	38 (67.9)	45 (73.8)	0.54
Diabetes (*n*, %)	24 (20.5)	11 (19.6)	13 (21.3)	1.0
CAD (*n*, %)	35 (29.9)	16 (28.6)	19 (31.1)	0.84
COPD (*n*, %)	51 (43.6)	28 (50.0)	23 (37.7)	0.19
Chronic renal failure (*n*, %)	27 (23.1)	12 (21.4)	15 (24.6)	0.82
Previous TIA/stroke (*n*, %)	17 (14.5)	7 (12.5)	10 (16.4)	0.60
PAD (*n*, %)	29 (24.8)	9 (16.1)	23 (37.7)	0.012
ASA score III-IV (*n*, %)	13 (11.1)	9 (16.1)	4 (6.6)	0.14

**Table 2:** Anatomical characteristics of the proximal aortic neck according to gender. AAA: abdominal aortic aneurysm; DT: transversal diameter.

	Overall Population (N. = 117)	Group A (Females) (N. = 56)	Group B (Males) (N. = 61)	*p*-Value
AAA DT max (mm ± SD, range)	56.7 ± 5.2	56.0 ± 5.6 (50–67)	57.4 ± 1.9 (50–78)	0.06
Neck length (mm ± SD, range)	15.3 ± 5.8	13.6 ± 5.0 (8–28)	16.9 ± 6.1 (8–32)	0.001
Neck diameter (mm ± SD, range)	24.2 ± 3.0	23.8 ± 3.1 (16–29)	24.6 ± 2.9 (17–29)	0.15
β-angle (° ± SD, range)	39.7 ± 16.3	42.8 ± 17.2 (17–86)	36.9 ± 15.0 (14–74)	0.051
Neck length < 10 mm (*n*, %)	23 (19.7)	17 (30.4)	6 (9.8)	0.009
Neck diameter > 28 mm (*n*, %)	8 (6.8)	6 (10.7)	2 (3.3)	0.15
β-angle > 60° (*n*, %)	16 (13.7)	12 (21.4)	4 (6.6)	0.029
Calcification/thrombosis > 50% (*n*, %)	12 (10.3)	7 (12.5)	5 (8.2)	0.54
Conical shape (*n*, %)	7 (6.0)	3 (5.4)	4 (6.6)	1.0
Associated hostile neck features (*n*, %)	35 (29.9)	25 (44.6)	10 (16.4)	0.001

**Table 3:** Type of stent grafts, rate of suprarenal fixation, and off-label use of stent grafts.

	Overall Population (N. = 117)	Group A (Females) (N. = 56)	Group B (Males) (N. = 61)	*p*-Value
Gore Excluder (*n*, %)	23 (19.7)	8 (14.3)	15 (24.6)	0.17
AFX2 (*n*, %)	25 (21.4)	8 (14.3)	17 (27.9)	0.11
Gore CXT (*n*, %)	24 (20.5)	14 (25)	10 (16.4)	0.26
Cook Zenith (*n*, %)	18 (15.4)	10 (17.9)	8 (13.1)	0.60
Endurant II/IIs (*n*, %)	14 (12.0)	8 (14.3)	6 (9.8)	0.57
Incraft (*n*, %)	1 (0.9)	1 (1.8)	0 (0)	0.47
Ovation/Alto (*n*, %)	12 (10.2)	7 (12.5)	5 (8.2)	0.54
Suprarenal fixation (*n*, %)	45 (38.5)	26 (46.4)	19 (31.1)	0.12
Off-label use (*n*, %)	23 (19.6)	16 (28.6)	7 (11.5)	0.034

**Table 4:** Outcomes of peri-operative and follow-up period.

**Peri-Operative**	**Overall Population** **(N. = 117)**	**Group A (Females)** **(N. = 56)**	**Group B (Males)** **(N. = 61)**	***p*** **-Value**
Technical success (*n*, %)	115 (98.3)	55 (98.2%)	60 (98.3%)	1.0
Type 1A endoleak (*n*, %)	2 (1.7)	1 (1.8)	1 (1.6)	1.0
Neck-related reintervention (*n*, %)	1 (9.4)	0 (0)	1 (1.6)	1.0
**Follow-up**	**Overall Population** **(N. = 106)**	**Group A (Females)** **(N. = 51)**	**Group B (Males)** **(N. = 55)**	***p*** **-Value**
Type 1A endoleak (*n*, %)	11 (10.4)	7 (13.7)	4 (7.3)	0.34
Neck-related reintervention (*n*, %)	11 (10.4)	7 (13.7)	4 (7.3)	0.34
Open conversion (*n*, %)	6 (5.7)	3 (5.9)	3 (5.4)	1.0
Aneurysm-related mortality (*n*, %)	4 (3.8)	2 (3.9)	2 (3.6)	1.0
**Off-label EVAR**	**Overall Population** **(N. = 23**)	**Group A (Females)** **(N. = 16)**	**Group B (Males)** **(N. = 7)**	***p*** **-Value**
Type 1A endoleak (*n*, %)	12 (52.2)	8 (50)	4 (57.1)	1.0
Neck-related reintervention (*n*, %)	11 (47.8)	7 (43.8)	4 (57.1)	0.66
Open conversion (*n*, %)	3 (13.0)	1 (6.2)	2 (28.6)	0.20
Aneurysm-related mortality (*n*, %)	4 (17.4)	2 (12.5)	2 (28.6)	0.55

## 4. Discussion

Since the advent of endovascular surgery, the feasibility and safety of EVAR in female patients have been consistently challenged by anatomical differences, including a narrower proximal aortic neck, smaller iliac artery diameter, and increased arterial tortuosity compared to male patients. These gender-related anatomical disparities significantly compromise the eligibility for standard EVAR procedures in women [17,18,19]. Evidence from the recent literature indicates that female patients are generally older at the time of EVAR intervention, often presenting with multiple comorbidities, higher ASA scores, and increased frailty [20], all of which contribute to an elevated surgical risk for open repair. Given their higher surgical risk profile, it would be expected that EVAR should be the preferred treatment option for women. However, these anatomical constraints not only limit the applicability of EVAR but are also associated with poorer early and late outcomes in female patients, including higher complication rates, reduced technical success, and increased reintervention rates [21]. Addressing these challenges requires advancements in device design, optimized preoperative planning, and a patient-specific approach to improve procedural outcomes and long-term success in women undergoing EVAR.

Notably, proximal aortic neck morphology is one of the primary limiting factors for EVAR eligibility [3,22]. In our study, female patients more frequently exhibited hostile aortic neck features compared to male patients (*p* = 0.001), despite having aneurysms of similar diameters (*p* = 0.06). This observation aligns with the existing literature, which highlights that short, angulated, and calcified proximal aortic necks are more prevalent in women, contributing to the reduced feasibility and technical success of EVAR in this population. However, it is important to note that many of these studies did not control for aneurysm size, potentially confounding the association between aortic neck morphology and gender-specific EVAR outcomes [7,22]. These gender differences in proximal neck anatomy may be attributable to a more pronounced aortic degenerative process and subsequent neck remodeling in women by the time the AAA reaches the threshold dimensions for intervention. This accelerated degenerative remodeling could result in hostile aortic neck features, which in turn compromise optimal stent graft sealing and fixation during and after EVAR.

It follows that women may experience higher rates of neck-related complications, reinterventions, open conversions, and mortality compared to men [23]. These outcomes are likely driven by the inherent anatomical challenges of the proximal aortic neck. Additionally, the smaller iliac artery diameter and increased vessel tortuosity frequently observed in women may further contribute to technical difficulties during stent graft deployment and an elevated risk of peri-procedural complications.

Interestingly, in our study, although women presented with shorter and more angulated aortic necks (*p* = 0.001 and *p* = 0.051, respectively), these anatomical differences did not significantly impact technical success (*p* = 1.0) and overall procedural outcomes when compared to men. This finding suggests that, despite the well-documented anatomical challenges in female patients, careful preoperative planning, appropriate device selection [24], and refined endovascular techniques may mitigate the negative effects of these unfavorable neck characteristics on procedural success and short-term outcomes. Although women experienced a higher incidence of type 1A endoleaks and neck-related reinterventions, these differences did not reach statistical significance (*p* = 0.34) and did not appear to compromise overall EVAR outcomes. This suggests that, despite the anatomical challenges, procedural success and early outcomes in women remain comparable to those observed in men.

These findings are consistent with recent studies reporting comparable EVAR outcomes between genders, despite the more complex aortic morphology in females [25,26].

This suggests that advances in stent graft technology, refined endovascular techniques, and meticulous preoperative planning may effectively mitigate the anatomical challenges typically encountered in women, leading to equivalent technical success rates and short-term outcomes [27,28]. However, continued investigation is warranted to assess whether these similarities persist in the long-term follow-up period.

In our study, females were more likely to undergo suprarenal fixation to achieve adequate sealing compared to men, although this difference did not reach statistical significance. This increased reliance on suprarenal fixation likely reflects the greater prevalence of associated hostile proximal neck anatomy in women (*p* = 0.001). Suprarenal fixation may serve as an effective strategy to partially overcome these anatomical limitations, ensuring improved stent graft stability and reducing the risk of Type 1A endoleaks.

Nonetheless, despite advancements in endograft technology, a hostile proximal aortic neck may still limit the on-label applicability of EVAR for some or even all currently available devices. Anatomical challenges often exceed the recommended device IFUs. As a result, off-label EVAR remains a common practice in these scenarios, potentially exposing patients to higher rates of complications, reinterventions, and reduced long-term durability.

In our series, a significantly higher proportion of women underwent off-label EVAR compared to men (*p* = 0.034). Although several studies have investigated the relationship between adherence to IFUs and EVAR outcomes [29,30], no clear consensus has been reached [31].

In our study, non-adherence to IFUs was associated with a higher incidence of type 1A endoleaks, neck-related reinterventions, and open conversions. However, univariate analysis revealed that the outcomes of off-label EVAR were not influenced by gender (Table 4). These findings suggest that while off-label EVAR remains a necessary approach in anatomically challenging cases and frail patients, its outcomes are more dependent on anatomical factors (e.g., severe neck angulation, conical shape) and procedural technique than on patient gender. Further research with larger cohorts and extended follow-up periods is needed to fully understand the long-term implications of off-label EVAR across different patient populations. Additionally, such studies are essential to confirm the absence of gender-specific differences in outcomes and clarify whether anatomical factors alone drive the observed results, irrespective of patient sex.

### Limitations

This study was a single-center retrospective cohort study. The main limitation of this study is the limited enrolled population (117 patients over a period of four years). Although the small size of the study population does not allow us to draw definitive conclusions, our experience suggests a gender-related tailored approach to stent graft selection. Aware of these limitations, this is one of the few studies that has evaluated the impact of sex-related differences for infrarenal aortic neck morphology on outcomes of patients undergoing endovascular aneurysm repair for similar-sized aortic aneurysms.

## 5. Conclusions

The proximal aortic neck anatomy in female patients appears to be significantly more hostile compared to that in male patients, even when aneurysm diameters are comparable. Our study confirms that the female cohort more frequently exhibits multiple hostile neck characteristics simultaneously, further reducing the feasibility of on-label standard EVAR procedures.

Additionally, female patients tend to undergo EVAR treatment at an older age compared to male patients. Despite these anatomical and demographic differences, no significant variation was observed in technical success rates or mid- to long-term outcomes of EVAR between genders. Furthermore, the distribution of stent graft types was evenly balanced across both cohorts, suggesting that procedural planning, proper device selection, and surgeons’ expertise may effectively mitigate the challenges posed by gender-related anatomical differences.

As a result, the historical outcome disparities associated with gender-specific anatomical differences have been progressively reduced, contributing to comparable technical success rates and mid- to long-term results between male and female patients.

Further and larger studies are essential to confirm the absence of gender-specific differences in outcomes and clarify whether anatomical factors alone drive the observed results, irrespective of patient sex.

## Data Availability

The data presented in this study are available on request from the corresponding author. The data are not available due to privacy restrictions.
